# A Comparative Analysis of Microbe-Based Technologies Developed at ICAR-NBAIM Against *Erysiphe necator* Causing Powdery Mildew Disease in Grapes (*Vitis vinifera* L.)

**DOI:** 10.3389/fmicb.2022.871901

**Published:** 2022-05-17

**Authors:** Deepti Malviya, Ratna Thosar, Namrata Kokare, Shital Pawar, Udai B. Singh, Sujoy Saha, Jai P. Rai, Harsh V. Singh, R. G. Somkuwar, Anil K. Saxena

**Affiliations:** ^1^Plant-Microbe Interaction and Rhizosphere Biology Lab, ICAR-National Bureau of Agriculturally Important Microorganisms, Maunath Bhanjan, India; ^2^ICAR-National Research Centre for Grapes, Pune, India; ^3^Department of Mycology and Plant Pathology, Institute of Agricultural Sciences, Banaras Hindu University, Varanasi, India

**Keywords:** microbe-based technology, Eco-Pesticide, Bio-Pulse, Bio-Care, *Erysiphe necator*, *Vitis vinifera*, powdery mildew of grapes

## Abstract

Globally, *Erysiphe necator* causing powdery mildew disease in grapevines (*Vitis vinifera* L.) is the second most important endemic disease, causing huge economic losses every year. At present, the management of powdery mildew in grapes is largely dependent upon the use of chemical fungicides. Grapes are being considered as one of the high pesticide-demanding crops. Looking at the residual impact of toxic chemical pesticides on the environment, animal, and human health, microbe-based strategies for control of powdery mildew is an emerging technique. It offers an environment-friendly, residue-free, and effective yet safer approach to control powdery mildew disease in grapes. The mode of action is relatively diverse as well as specific to different pathosystems. Hence, the aim of this study was to evaluate the microbe-based technologies, i.e., Eco-pesticide^®^, Bio-Pulse^®^, and Bio-Care 24^®^ developed at the Plant-Microbe Interaction and Rhizosphere Biology Lab, ICAR-NBAIM, Kushmaur, against grape powdery mildew and to integrate these technologies with a safer fungicide (sulfur) to achieve better disease control under organic systems of viticulture. The experiments were conducted at four different locations, namely, the vineyards of ICAR-NRCG, Rajya Draksha Bagayatdar Sangh (MRDBS), and two farmers' fields at Narayangaon and Junnar in the Pune district of Maharashtra. A significantly lower percent disease index (PDI) was recorded on the leaves of grape plants treated with Eco-Pesticide^®^/sulfur (22.37) followed by Bio-Pulse^®^/sulfur (22.62) and Bio-Care 24^®^/sulfur (24.62) at NRCG. A similar trend was observed with the lowest PDI on bunches of Eco-pesticide^®^*/*sulfur-treated plants (24.71) followed by Bio-Pulse^®^/sulfur (24.94) and Bio-Care^®^/sulfur (26.77). The application of microbial inoculants singly or in combination with sulfur has a significant positive impact on the qualitative parameters such as pH, total soluble solids (TSS), acidity, berry diameter, and berry length of the grapes at different locations. Among all the treatments, the Bio-Pulse^®^/sulfur treatment showed the highest yield per vine (15.02 kg), which was on par with the treatment Eco-Pesticide^®^/sulfur (14.94). When compared with the yield obtained from the untreated control, 2.5 to 3 times more yield was recorded in the plants treated with either of the biopesticides used in combination with sulfur. Even in the case of individual inoculation, the yield per vine was approximately two times higher than the untreated control and water-treated plants across the test locations. Results suggested that microbial technologies not only protect grapevines from powdery mildew but also enhance the quality parameters with increased yield across the test locations.

## Introduction

Grapevine (*Vitis vinifera* L.) is one of the important crops grown worldwide for wine, dried resins, and fresh table purposes. It was originally a temperate crop but is widely cultivated in temperate, subtropical, and tropical regions of the world. Several reports indicate that ~72 million tons of grapes are produced worldwide every year, most of which are used to produce wine. Apart from wine production, grapes are widely used to prepare jelly, jam, juices, raisins, currants, and sultanas (Sawant and Sawant, 2006; Sawant et al., 2017). It has great economic potential due to higher yields translating into higher monetary returns, which are duly supported by its fair export potential (Calonnec et al., 2004). Being an export crop, it plays a crucial role in the nation's economy. In India, it is widely cultivated in the states of Maharashtra, Karnataka, Tamil Nadu, Mizoram, and Andhra Pradesh. The area under grapes in India is ~1.25 lakh hectares with an average productivity of 22.95 t/ha. Among these states, Maharashtra contributes about 75.85% to the area and 81.22% to the national grape production with a productivity of 24.58 t/ha (Sawant and Sawant, 2006; Sawant et al., 2017; Kanitkar et al., 2020).

Several biotic (viruses, bacteria, fungi, and insects) and abiotic (i.e., drought and winter cold) stresses affect grape production worldwide. Among biotic stresses, fungal diseases, namely, downy mildew (*Plasmopara viticola* [Berk and Curtis] Berlese and De toni), powdery mildew (*Erysiphe necator* previously known as *Uncinula necator* [Schw.] Burn), and Anthracnose (*Gloeosporium ampelophagum* [Pass] Sacc. [Perfect stage: *Elsinoe ampelina* {DeB} Shear]) are the major constraints in grapevine cultivation (Calonnec et al., 2004; Gadoury et al., 2007, 2012; Vinothini et al., 2014). Among fungal diseases, powdery mildew is the second most important endemic disease of commercial grapevine varieties after downy mildew, and it becomes more serious than downy mildew in the changing climatic scenario with relatively cool and dry weather (Calonnec et al., 2004, 2018; Bendek et al., 2007). *Erysiphe necator* is an obligate biotrophic and the most notorious pathogen of the grapevine causing considerable losses in grape production (Konstantinidou-Doltsinis et al., 2007; Saleh et al., 2007). The disease can be devastating to susceptible varieties under conducive environmental conditions covering the entire above-ground parts of the plants. The release of ascospores is always associated with high humidity, and therefore, frequent rain is a key factor for the release of ascospores, which are, in fact, the primary inocula (Jones et al., 2014; Sawant et al., 2017; Kavadia et al., 2020). Grapevine diseases can have drastic ill effects not only on the host plants and berries but also on the wine qualities and their sensorial and organoleptic properties (Stummer et al., 2003a,b; Pinar et al., 2017a,b), resulting in economic losses for the grape growers and wine producers (van Helden, 2008). As a consequence of smaller diseased berries, *E. necator* can cause a drastic reduction in grape yield of up to 45% (Calonnec et al., 2004) and severally affect the export quality (Stummer et al., 2005; Rusjan et al., 2012; Pinar et al., 2016, 2017a,b). Although the grapevine is susceptible to powdery mildew at all its growth stages, berries are not infected after the berry softening stage (Calonnec et al., 2004, 2018; Gadoury et al., 2007, 2012).

Management of powdery mildew in grapes is largely dependent upon the use of chemical fungicides, and interestingly, grapes are considered to be one of the high pesticide-demanding crops (Sholberg et al., 2006; Pertot et al., 2017; Arestova and Ryabchun, 2021). Worldwide, an average of 35% of all pesticides produced are used in viticulture (Essling et al., 2021). In India, a total of 1,814 M.T. of pesticides were used in fruit crop production during 2020–2021 (www.ppqs.Gov.in). Earlier, sulfur and sulfur-containing fungicides were used for controlling the powdery mildew of grapes globally (Biondi et al., 2012; Warneke et al., 2022). However, in the recent past, several other fungicides, namely, difenoconazole, metrafenone, nissodium fenarimol, bupirimate, penconozole, dimethomorph, triademefon, pyrazophos, hexaconazole, chlorothalonil, and flusilazole were introduced in India and used to control powdery mildew in grapes (Sawant and Sawant, 2006; Sawant et al., 2017; Kanitkar et al., 2020). Consequences of intensive pesticide use include their persistence in soils, contamination of the environment, negative impact on human health, and deterrents to the ecosystems as well as the development of resistant pathogenic strains. Heavy doses and multiple applications of fungicides on grapes lead to excess fungicidal residues in the harvest, which affect the export quality and cause huge losses in foreign exchange (Carisse et al., 2009; Alem et al., 2019; Rantsiou et al., 2020). Resistance development in the pathogens and residual toxicity of chemical fungicides on the environment and human health have compelled researchers and commercial grape growers to look for alternative strategies (Yildirim and Dardeniz, 2010; Miles et al., 2012; Fernández-González et al., 2013; Çetinkaya and Fadime, 2016). With the possible withdrawal of chemical fungicides, including sulfur powder, from the schedule of the acceptable input chart and the demand for residue-free grapes, there is an urgent need to find suitable alternatives for disease management in the organic systems of viticulture (Carisse et al., 2009; Yildirim and Dardeniz, 2010; Lu H. et al., 2020). Among them, the development of resistant cultivars with a high degree of resistance/tolerance to respective pathogens to produce high-quality grapes and wines commensurate with the parameters for higher standards of food safety is of great importance (Pap et al., 2016; Riaz et al., 2020). However, the detection of the source of resistance to *Erysiphe necator* and the transfer of desired traits into a suitable commercial cultivar using a resistance breading program is a great challenge to the grape breeders (Ficke et al., 2002; Riaz et al., 2013, 2020; Pap et al., 2016). Furthermore, availability of resistant lines and breeding of resistant cultivars is cost-effective, but in grapes, it is not an easy task (Miles et al., 2012; Fernández-González et al., 2013; Çetinkaya and Fadime, 2016).

Under these circumstances, the use of microbe-based strategies for control of powdery mildew is an emerging technique/approach. It has been reported to be an environment-friendly, residue-free, and safer approach for combating the powdery mildew pathogen effectively (Hayes, 2015; Kumar et al., 2021; Pathma et al., 2021; Sellitto et al., 2021). In the recent past, several biological control agents of microbial origin have been evaluated and used to control the powdery mildew pathogen in grapes. Among them, *Ampelomyces quisqualis, Trichoderma harzianum, T. asperellum, T. virens, Pythium oligandrum, Pseudozyma flocculosa, Bacillus subtilis, B. licheniformis, B. brevis, B. cereus, Pseudomonas fluorescens*, and *Streptomyces cacaoi* were noteworthy (Rao et al., 2015; Damalas and Koutroubas, 2018; Thakur et al., 2020; Salimi and Hamedi, 2021). However, very few microbe-based products/technologies are available in the market for wider applicability in the Indian subcontinent and abroad to control grape powdery mildew in the organic viticulture (Compant et al., 2013; Moyer et al., 2016; Cangi et al., 2018; Malićanin et al., 2020). Due to a lack of information in the scientific literature on the availability and effectivity of microbial inoculants, agronomists and vine growers are often not aware of these new products and the impact they can have indirectly on the quality of grapes (Lu W. et al., 2020; Agbowuro et al., 2021; Steiner et al., 2021).

Recently, a few biopesticides of microbial origin have been developed at the Plant-Microbe Interaction and Rhizosphere Biology Lab, ICAR-National Bureau of Agriculturally Important Microorganisms (ICAR-NBAIM), Kushmaur, Maunath Bhanjan, Uttar Pradesh, India. Among them, Eco-pesticide^®^ (a liquid bioformulation of *Pseudomonas fluorescens* PF-08), Bio-Pulse^®^ (a talc-based bioformulation of *Trichoderma asperellum* UBSTH-501 and *Bacillus amyloliquefaciens* B-16), and Bio-Care 24^®^ (a liquid bioformulation of *Bacillus subtilis* RP-24) are widely studied technologies in different crops for enhanced resistance to biotic and abiotic stresses through direct and indirect mechanisms (Singh et al., 2016a,b, 2019a,b). The direct mechanism includes mycoparasitism, synthesis of many secondary metabolites, hormones, cell wall-degrading enzymes, and antioxidants that assist the plant in its defense against pathogenic attack (Singh et al., 2016a,b, 2019a,b). They were also found to increase plant growth, uptake, and translocation of the key plant nutrients from the soil, and thus increase yield directly and/or indirectly in many crops (Singh et al., 2016a,b, 2021). Keeping this in mind and analyzing the importance of grapes as an economical crop, trials were devised in collaboration with ICAR-NRC for Grapes, Pune, to evaluate and compare the (1) efficacy of microbe-based technologies, Eco-pesticide^®^, Bio-Pulse^®^, and Bio-Care 24^®^ developed at ICAR-NBAIM against grape powdery mildew and (2) integration of microbe-based technologies with the safer fungicide (sulfur) to achieve the better disease control with reduced fungicide application for wider applicability under organic viticulture.

## Materials and Methods

### Source of Microbe-Based Technologies

Eco-pesticide^®^, Bio-Pulse^®^, and Bio-Care 24^®^ were developed and supplied by the Plant-Microbe Interaction and Rhizosphere Biology Lab, ICAR-NBAIM, Kushmaur, Maunath Bhanjan, India. The colony-forming units (CFU) of Eco-pesticide^®^ (2.75 × 10^8^ ml^−1^), Bio-Pulse^®^ (*T. asperellum*: 2.25 × 10^7^ g^−1^, and *B. amyloliquefaciens*: 2.50 × 10^8^ g^−1^), and Bio-Care 24^®^ (3.50 × 10^8^ ml^−1^) were standardized before final packaging of the product.

### Experimental Setup

The experimental trials were conducted at four different locations, namely, vineyards of ICAR-National Research Center for Grapes (ICAR-NRCG), Pune (location 18.32°N, 73.51°E, soil black [Vertisol], with a pH of 7.75), Maharashtra, Rajya Draksha Bagayatdar Sangh (MRDBS), Pune (location 18.32°N, 73.51°E, soil slightly alkaline with a pH of 8.05 with climate hot semiarid climate bordering with tropical wet and dry and having average temperatures ranging from 66°F to 91°F), and two farmers' plots at Narayangaon and Junnar (19.2°N 73.88°E, temperature typically varies from 53°F to 96°F and is rarely below 47°F or above 100°F, soil black, Vertisol with a pH of 6.75; the wet season is warm, oppressive, windy, and overcast, and the dry season is hot and mostly clear) in the Pune district of Maharashtra. The cultivar Fantasy Seedless was taken for experimentation at ICAR-NRCG, Pune. However, a vineyard with the cultivar Nanasaheb Purple was taken into study in the other three locations, namely, MRDBS, a farmers' plot at Narayangaon and Junnar. The vines selected for the experiment were subjected to natural infection of powdery mildew. The first spray was carried when the disease infection was observed in the untreated control plot. The experiments were laid out in randomized block design (RBD) with four replications having eight grapevines per treatment. Grapevines sprayed with sulfur (80% WDG) were used as the standard check. The bio-formulations, which were taken for experimentation, were also applied with the alternation of sulfur (80% WDG). The main reasons behind alternate application of sulfur along with microbial technologies are that “only bioinoculants may not be sufficient to control the menace of obligate pathogens like powdery mildew and a need-based application of sulfur fungicide is needed.” The schedule of applications is given in Table 1. These microbial bio-pesticides were applied at weekly intervals. The volume of water used for spray was calculated (1,000 L/ha at full canopy). A knapsack sprayer with a hollow cone nozzle was used for spraying.

**Table 1:** Details of treatments for powdery mildew field trial.

**Treatments**	**Treatment details**	**Dose (ml or g/liter)**
T1	Eco-Pesticide	10 ml/l
T2	Bio-Pulse	10 g/l
T3	Bio-Care 24	10 ml/l
T4	Three sprays of Eco-Pesticide/one spray of sulfur	10 ml/l
T5	Three sprays of Bio-Pulse/one spray of sulfur	10 g/l
T6	Three sprays of Bio-Care 24/one spray of sulfur	10 ml/l
T7	Sulfur 80%WDG	2 gm/l
T8	Water control	-
T9	Untreated control	-

### Sampling and Analyses

#### Foliar Disease Intensity

**Figure 1:**
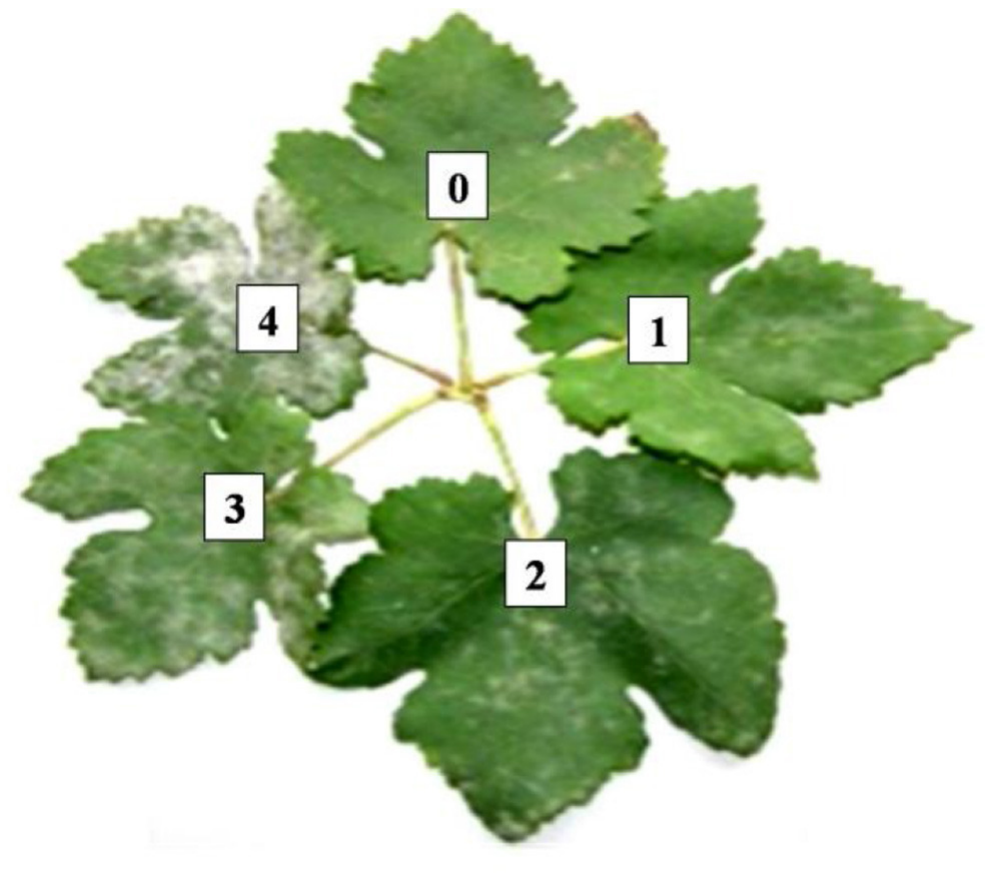
Pictorial depiction of 0–4 rating scale for powdery mildew disease severity.

The severity of powdery mildew was recorded at two different growth stages, first on leaves and second on bunches. The severity of powdery mildew on plant leaves was recorded by adopting the 0–4 scale, where 0 means no disease present and 4 means more than 75% of the leaf area is infected. A rating scale on leaves is shown in Figure 1. PDI was calculated using the following formula:


$$\begin{array}{l} \text{PDI}=\;\frac{\text{Sum of numerical ratings }\times \;100}{\text{Number of leaves observed }\times \text{ Maximum rating scale}} \end{array}$$

The ratings on 10 leaves were recorded on randomly selected canes. Such 10 canes per vine were observed, so 100 diseased leaf observations were recorded per replicate. Four replications for each treatment were considered. Only actively growing powdery mildew lesions were considered for recording ratings.

#### Bunch Infection

During the fruiting season, powdery mildew ratings were recorded separately on bunches. Powdery mildew appearance on bunches was recorded by adopting a 0–4 scale, where 0 means no disease present and 4 means more than 75% of the bunch area is infected. PDI was calculated using the following formula:


$$\begin{array}{l} \text{PDI}=\;\frac{\text{Sum of numerical ratings }\times \;100}{\text{Number of bunches observed }\times \text{ Maximum rating scale}} \end{array}$$

The ratings on 20 randomly selected bunches per replicate were recorded. During observations, only active powdery mildew growth was considered for recording ratings.

#### Estimation of TSS, Titrable Acidity, pH, and Physiological Loss in Weight

Fruits from different treatments were harvested and used for the analysis of various fruit quality, qualitative, and quantitative parameters, namely, total soluble solids (TSS), titratable acidity (TA), pH, physiological weight loss (PWL), and marketable yield. The total soluble solids (TSS) and titratable acidity (TA) were estimated by extracting juice from crushed berries and centrifuging at 5,000 rpm for 5 min. TSS was estimated using the digital handheld refractometer with a temperature compensated to 20°C (Thosar et al., 2020). Determination of titratable acidity was conducted by titration with 0.1N of NaOH using phenolphthalein as the indicator and titratable acidity was expressed as tartaric acid equivalent (Satisha and Somkuwar, 2019).

Percentage acid = Titer × acid factor × 10/10 (ml juice)

where “factor” for grapefruit is 0.075 (Satisha and Somkuwar, 2019).

The pH of the juice was recorded using a pH meter (Model 420, Thermo Orion) as per the methods described by Satisha and Somkuwar (2019). The physiological weight loss of berries was also assessed. The weight of bunches was recorded at 24-h intervals for the first 5–7 days at room temperature. The percentage of weight loss over the initial weight was calculated mathematically (Thosar et al., 2020).

#### Marketable Yield

To calculate the total marketable yield, fruits were harvested from each treatment in four replications, including the untreated control plants, and the yield was calculated in kg/ha.

### Statistical Analysis

The PDI data were transformed using an arcsine transformation for leaves and bunches and statistically analyzed using a randomized block design (RBD) using the Statistical Analysis System (SAS software, version 9.3). The yield data were analyzed without transformation. Means were compared using the least significant difference (LSD) test.

## Results

### Effect of Microbial Bioformulations on the Severity of Powdery Mildew on Leaves

The first disease symptom was recorded in the experimental plot on 24 December 2020 in the untreated control (Table 2). Results indicated that significantly less disease (PDI) was recorded in the plant leaves treated with either of the microbial inoculants individually or in combination with sulfur (80% WDG) as compared to the untreated control plants (37.78) and water-treated plants (29.57) grown at ICAR-NRCG, Pune. However, the least disease (PDI) was recorded on the leaves of plants treated with fungicide (sulfur 80% WDG) on 29 January 2021 (PDI: 21.07). Among different microbial inoculations, a significantly lower disease index (PDI) was recorded on the leaves of grape plants treated with Eco-Pesticide^®^/sulfur (22.37) followed by Bio-Pulse^®^/sulfur (22.62) and Bio-Care 24^®^/sulfur (24.62). Moreover, the last four observations recorded between 8 January 2021 and 29 January 2021 indicated that powdery mildew was significantly higher in the untreated control than in all the other treatments with microbial inoculation. Bio-Pulse^®^/sulfur and Eco-Pesticide^®^/sulfur (at 10 ml L^−1^) were statistically on par with each other. The trend was similar during the first, second, third, and fourth observations also (Table 2). Looking at the individual treatments, the least PDI was observed in the plants treated with Eco-Pesticide^®^ (25.91) followed by Bio-Pulse^®^ (26.09) and Bio-Care 24^®^ (28.04) as compared to the untreated control plants (37.78) and water-treated plants (29.57) at ICAR-NRCG (Table 2). The data in Table 2 clearly indicate that maximum PDI was recorded in the untreated control plants followed by water-treated plants, while the least PDI was observed in the plants treated with sulfur (80% WDG) across the locations. Results indicated that comparatively less disease was recorded on the leaves of plants treated with Bio-Pulse^®^/sulfur followed by Eco-Pesticide^®^/sulfur and Bio-Care 24^®^/sulfur at MRDBS and farmers' plots at Narayangaon and Junnar in the Pune district of Maharashtra (Table 2). However, relatively higher PDI was observed on the leaves of untreated control plants grown at MRDBS, followed by farmers' plots at Narayangaon and Junnar, Pune, as compared to ICAR-NRCG (Table 2).

**Table 2:** Bio-efficacy of biocontrol agent formulations against powdery mildew of grapes at different locations.

**Treatments**	**PDI of powdery mildew on leaves**				
**At ICAR-NRCG, Pune**	**24/12/2020**	**08/01/2021**	**14/01/2021**	**22/01/2021**	**29/1/2021**
T_1_-Eco-Pesticide	0.00 (0.00)	12.75 (20.90)	14.81 (22.62)	17.06 (24.38)	19.12 (25.91)
T_2_-Bio-Pulse	0.00 (0.00)	13.00 (21.12)	15.13 (22.87)	17.25 (24.48)	19.37 (26.09)
T_3_-Bio-Care 24	0.00 (0.00)	15.81 (23.41)	17.94 (25.04)	20.06 (26.59)	22.13 (28.04)
T_4_-Eco-Pesticide/sulfur	0.00 (0.00)	8.13 (16.50)	10.25 (18.66)	12.38 (20.58)	14.50 (22.37)
T_5_-Bio-Pulse/sulfur	0.00 (0.00)	8.38 (16.81)	10.50 (18.89)	12.63 (20.80)	14.81 (22.62)
T_6_-Bio-Care 24/sulfur	0.00 (0.00)	10.94 (19.30)	13.13 (21.23)	15.25 (22.97)	17.37 (24.62)
T_7_-Sulfur 80%WDG	0.00 (0.00)	6.56 (14.83)	8.69 (17.13)	10.81 (19.18)	12.93 (21.07)
T_8_-Water control	0.00 (0.00)	18.06 (25.12)	20.12 (26.64)	22.25 (29.87)	24.38 (29.57)
T_9_-Untreated control	3.56 (10.85)	24.81 (29.90)	29.06 (32.60)	33.14 (35.13)	37.56 (37.78)
**CD (*****P** **=*** **0.05)**	**0.39**	**0.75**	**0.65**	**1.87**	**0.73**
**At MRDBS, Pune**	**20/12/2020**	**08/01/2021**	**14/01/2021**	**22/01/2021**	**29/1/2021**
T_1_-Eco-Pesticide	0.00 (0.00)	14.75 (22.56)	17.25 (24.47)	19.81 (26.41)	23.75 (29.15)
T_2_-Bio-Pulse	0.00 (0.00)	15.06 (22.81)	17.75 (24.80)	20.13 (26.63)	24.06 (29.36)
T_3_-Bio-Care 24	0.00 (0.00)	19.25 (25.99)	19.75 (27.20)	22.88 (28.55)	26.75 (31.13)
T_4_-Eco-Pesticide/sulfur	0.00 (0.00)	8.75 (17.12)	13.25 (21.23)	13.88 (21.85)	17.75 (24.90)
T_5_-Bio-Pulse/sulfur	0.00 (0.00)	8.25 (16.67)	12.25 (21.33)	13.38 (21.41)	17.25 (24.51)
T_6_-Bio-Care 24/sulfur	0.00 (0.00)	11.31 (19.58)	14.25 (23.32)	17.25 (24.52)	20.31 (26.76)
T_7_-Sulfur 80%WDG	0.00 (0.00)	3.06 (10.01)	6.25 (15.65)	9.69 (18.10)	13.56 (21.58)
T_8_-Water control	0.00 (0.00)	28.88 (32.48)	30.00 (34.39)	33.94 (35.61)	37.88 (37.96)
T_9_-Untreated control	4.94 (12.82)	36.75 (37.30)	40.75 (39.42)	43.38 (41.17)	47.25 (43.40)
**CD (*****P** **=*** **0.05)**	**0.27**	**1.93**	**1.28**	**0.88**	**1.27**
**At Narayangaon, Pune**	**21/12/2020**	**09/01/2021**	**15/01/2021**	**23/01/2021**	**30/01/2021**
T_1_-Eco-Pesticide	0.00 (0.00)	13.25 (21.32)	16.31 (23.79)	18.81 (25.69)	22.75 (28.47)
T_2_-Bio-Pulse	0.00 (0.00)	13.56 (21.58)	16.63 (24.03)	19.13 (25.91)	23.06 (26.77)
T_3_-Bio-Care 24	0.00 (0.00)	16.25 (23.71)	19.31 (26.04)	22.50 (28.30)	25.75 (30.48)
T_4_-Eco-Pesticide/sulfur	0.00 (0.00)	7.25 (15.51)	10.31 (18.64)	12.88 (21.00)	16.75 (24.14)
T_5_-Bio-Pulse/sulfur	0.00 (0.00)	6.75 (15.04)	9.81 (18.16)	12.38 (20.56)	16.25 (23.75)
T_6_-Bio-Care 24/sulfur	0.00 (0.00)	10.00 (18.41)	12.06 (20.31)	15.06 (22.82)	19.13 (25.91)
T_7_-Sulfur 80%WDG	0.00 (0.00)	3.06 (10.01)	6.13 (14.30)	8.69 (17.11)	12.56 (20.74)
T_8_-Water control	0.00 (0.00)	22.50 (28.30)	30.43 (33.46)	32.94 (34.93)	36.88 (37.37)
T_9_-Untreated control	4.44 (12.14)	27.88 (31.36)	39.81 (39.09)	42.38 (41.38)	46.25 (42.83)
**CD (*****P** **=*** **0.05)**	**0.28**	**1.87**	**2.20**	**1.38**	**1.25**
**At Junnar, Pune**	**21/12/2020**	**09/01/2021**	**15/01/2021**	**23/01/2021**	**30/01/2021**
T_1_-Eco-Pesticide	0.00 (0.00)	13.25 (21.32)	15.81 (23.40)	18.31 (25.32)	24.88 (29.90)
T_2_-Bio-Pulse	0.00 (0.00)	13.56 (21.58)	16.13 (23.64)	18.63 (25.54)	25.25 (30.15)
T_3_-Bio-Care 24	0.00 (0.00)	16.25 (23.71)	18.81 (25.68)	21.38 (27.52)	28.00 (31.93)
T_4_-Eco-Pesticide/sulfur	0.00 (0.00)	7.25 (15.51)	11.88 (20.12)	12.38 (20.57)	18.81 (25.68)
T_5_-Bio-Pulse/sulfur	0.00 (0.00)	6.75 (15.03)	11.50 (19.80)	11.88 (20.05)	18.00 (25.07)
T_6_-Bio-Care 24/sulfur	0.00 (0.00)	9.81 (18.16)	13.88 (21.85)	15.75 (23.36)	19.00 (25.78)
T_7_-Sulfur 80%WDG	0.00 (0.00)	3.06 (10.01)	5.63 (13.69)	8.19 (16.59)	12.06 (20.31)
T_8_-Water control	0.00 (0.00)	27.38 (31.53)	29.93 (33.14)	32.44 (34.70)	36.38 (37.07)
T_9_-Untreated control	3.44 (10.66)	36.75 (37.30)	39.31 (38.80)	41.88 (40.30)	45.75 (42.54)
**CD (*****P** **=*** **0.05)**	**0.32**	**2.15**	**1.71**	**1.41**	**1.63**

*Figures in parenthesis indicate arcsine transformed averages*.

These data demonstrated that microbial inoculants not only impede initial infection of the powdery mildew pathogen, *E. necator*, on the plant leaves but also inhibit their invasion, colonization, and development, indicating that these biopesticides are strong inhibitors of *E. necator* along with being strong inducers of plant defense against powdery mildew pathogen in grapes.

### Effect of Microbial Bioformulations on Severity of Powdery Mildew on Bunches

Data in Table 3 show a significant difference between powdery mildew developed on bunches of the untreated control plants as compared to biopesticides-treated plants. In line with this observation, disease development in bunches was significantly reduced in biopesticides-treated plants as compared to the untreated control and water-treated plants across the locations. Furthermore, a delay and slow disease development were seen in biopesticide-treated plants, and the majority of cleistothecia were produced on bunches of the control plants, while significantly lesser cleistothecia were seen on bunches of the microbe-inoculated plants (data not shown). In the case of disease development on bunches, a similar trend was observed and the lowest PDI was recorded on bunches of Eco-pesticide^®^*/*sulfur-treated plants (24.71) followed by Bio-Pulse^®^/sulfur (24.94) and Bio-Care^®^/sulfur (26.77) as compared to the untreated control (39.94) and water-treated plants (31.60) at ICAR-NRCG. However, PDI of sulfur (80% WDG) was the lowest as compared to all the other treatments (23.70). A more or less similar trend was recorded at MRDBS and farmers' plot at Junnar in the Pune district of Maharashtra (Table 3).

**Table 3:** Bio-efficacy of biocontrol agent formulations against powdery mildew of grapes at different locations.

**Treatments**	**PDI of powdery mildew on bunches**				
**At ICAR-NRCG, Pune**	**24/12/2020**	**08/01/2021**	**14/01/2021**	**22/01/2021**	**29/1/2021**
T_1_-Eco-Pesticide	0.00 (0.00)	15.63 (23.27)	17.81 (22.90)	19.68 (26.32)	22.19 (28.08)
T_2_-Bio-Pulse	0.00 (0.00)	15.94 (23.51)	18.12 (25.17)	20.00 (26.54)	22.50 (28.30)
T_3_-Bio-Care 24	0.00 (0.00)	18.75 (25.64)	21.25 (27.43)	23.12 (28.72)	25.00 (29.98)
T_4_-Eco-Pesticide/sulfur	0.00 (0.00)	10.94 (19.28)	13.12 (21.20)	15.31 (23.00)	17.50 (24.71)
T_5_-Bio-Pulse/sulfur	0.00 (0.00)	11.25 (19.57)	13.43 (21.48)	15.62 (23.25)	17.81 (24.94)
T_6_-Bio-Care 24/sulfur	0.00 (0.00)	13.75 (21.69)	15.93 (23.50)	18.12 (25.18)	20.31 (26.77)
T_7_-Sulfur 80%WDG	0.00 (0.00)	9.37 (17.77)	11.56 (19.85)	13.75 (21.72)	15.63 (23.70)
T_8_-Water control	0.00 (0.00)	20.93 (27.21)	24.06 (29.35)	26.25 (30.76)	27.5 (31.60)
T_9_-Untreated control	4.18 (11.77)	27.81 (31.80)	31.87 (34.35)	36.62 (37.20)	41.25 (39.94)
**CD (*****P** **=*** **0.05)**	**0.46**	**1.66**	**2.04**	**1.45**	**0.48**
**At MRDBS, Pune**	**30/12/2020**	**08/01/2021**	**14/01/2021**	**22/01/2021**	**29/1/2021**
T_1_-Eco-Pesticide	0.00 (0.00)	16.25 (23.74)	10.31 (19.09)	26.25 (30.80)	29.06 (32.60)
T_2_-Bio-Pulse	0.00 (0.00)	16.88 (24.21)	9.69 (18.10)	24.69 (29.77)	28.13 (32.21)
T_3_-Bio-Care 24	0.00 (0.00)	22.81 (28.50)	24.06 (29.34)	30.31 (33.38)	33.75 (35.50)
T_4_-Eco-Pesticide/sulfur	0.00 (0.00)	7.81 (16.18)	19.69 (26.32)	18.75 (25.64)	18.13 (25.17)
T_5_-Bio-Pulse/sulfur	0.00 (0.00)	5.63 (15.01)	19.38 (26.10)	15.63 (24.22)	19.38 (26.07)
T_6_-Bio-Care 24/sulfur	0.00 (0.00)	13.13 (21.18)	14.69 (22.51)	22.50 (28.30)	23.44 (28.91)
T_7_-Sulfur 80%WDG	0.00 (0.00)	4.06 (11.60)	7.81 (16.21)	11.56 (19.74)	12.50 (20.68)
T_8_-Water control	0.00 (0.00)	27.81 (31.81)	31.56 (34.16)	39.06 (38.66)	41.88 (40.30)
T_9_-Untreated control	7.81 (16.16)	36.88 (37.36)	39.69 (39.02)	48.44 (44.26)	47.81 (43.72)
**CD (*****P** **=*** **0.05)**	**0.80**	**1.77**	**1.47**	**1.64**	**1.82**
**At Narayangaon, Pune**	**21/12/2020**	**09/01/2021**	**15/01/2021**	**23/01/2021**	**30/01/2021**
T_1_-Eco-Pesticide	0.00 (0.00)	8.13 (16.49)	13.12 (20.39)	15.94 (23.50)	22.19 (28.08)
T_2_-Bio-Pulse	0.00 (0.00)	8.44 (16.87)	12.81 (20.96)	14.38 (22.22)	21.56 (27.65)
T_3_-Bio-Care 24	0.00 (0.00)	14.06 (21.78)	18.12 (25.17)	23.13 (28.72)	26.88 (31.19)
T_4_-Eco-Pesticide/sulfur	0.00 (0.00)	5.63 (13.65)	8.75 (17.17)	10.31 (18.71)	15.63 (23.23)
T_5_-Bio-Pulse/sulfur	0.00 (0.00)	5.00 (12.86)	8.12 (16.54)	9.38 (17.79)	15.31 (22.98)
T_6_-Bio-Care 24/sulfur	0.00 (0.00)	10.63 (18.92)	15.31 (22.98)	16.87 (24.24)	18.13 (25.17)
T_7_-Sulfur 80%WDG	0.00 (0.00)	3.12 (10.04)	5.31 (13.30)	6.25 (14.43)	11.25 (19.39)
T_8_-Water control	0.00 (0.00)	28.43 (32.21)	31.87 (34.35)	34.06 (35.68)	38.44 (38.29)
T_9_-Untreated control	2.81 (2.81)	37.50 (37.74)	40.63 (39.57)	41.25 (39.94)	45.94 (42.65)
**CD (*****P** **=*** **0.05)**	**0.50**	**2.52**	**1.68**	**1.66**	**2.39**
**At Junnar, Pune**	**21/12/2020**	**09/01/2021**	**15/01/2021**	**23/01/2021**	**30/01/2021**
T_1_-Eco-Pesticide	0.00 (0.00)	16.25 (23.74)	8.75 (17.48)	24.06 (29.35)	27.50 (31.61)
T_2_-Bio-Pulse	0.00 (0.00)	16.88 (24.24)	8.13 (16.51)	24.69 (29.77)	28.13 (32.01)
T_3_-Bio-Care 24	0.00 (0.00)	21.25 (27.42)	22.50 (28.27)	28.43 (32.19)	32.19 (34.55)
T_4_-Eco-Pesticide/sulfur	0.00 (0.00)	6.25 (14.43)	18.13 (25.17)	16.87 (24.24)	16.25 (23.76)
T_5_-Bio-Pulse/sulfur	0.00 (0.00)	5.63 (13.87)	18.75 (25.64)	15.6 (23.27)	17.73 (24.89)
T_6_-Bio-Care 24/sulfur	0.00 (0.00)	11.56 (19.82)	13.13 (21.17)	20.62 (26.97)	21.69 (27.74)
T_7_-Sulfur 80%WDG	0.00 (0.00)	4.06 (11.60)	6.25 (14.43)	10.00 (18.18)	13.25 (21.33)
T_8_-Water control	0.00 (0.00)	27.81 (31.81)	29.69 (33.01)	37.5 (37.74)	40.31 (39.39)
T_9_-Untreated control	5.00 (12.86)	36.88 (37.36)	38.13 (38.11)	40.63 (39.57)	46.56 (43.01)
**CD (*****P** **=*** **0.05)**	**0.66**	**1.53**	**1.96**	**2.31**	**1.26**

*Figures in parenthesis indicate arcsine transformed averages*.

In contrast, PDI of Bio-Pulse^®^/sulfur-treated bunches (22.98) was the lowest followed by Eco-pesticide^®^*/*sulfur-treated plants (23.23) and Bio-Care^®^/sulfur (25.17) (both statistically on par with each other) as compared to the untreated control (42.65) at farmers' plot, Narayangaon, Pune. The trend was also similar during the first, second, third, and fourth observations (Table 3).

### Effect of Microbial Bioformulations on the Shelf Life of Grape Bunches

The shelf life of grape bunches is one of the most important attributes for grape export quality. The longer shelf life of the grape bunches facilitates the grapes' longer distance transportation by keeping their market value and good appearance unabated. Therefore, the effect of bioformulation application on the shelf life of bunches was recorded. The shelf life of bunches was estimated by keeping the harvested bunches at room temperature and recording the loss in bunch weight at 24 h of intervals. With increasing the storage duration, the physiological loss in weight (PLW) was also increased. In general, among all the treatments, microbial biopesticides in combination with sulfur manifested lesser PLW as compared to the untreated control and water-treated bunches. To determine whether microbial inoculants, individually or in combination with sulfur, were involved in the PLW, berry rotting, and berry dropping directly and/or indirectly, observations on shelf life were recorded at different time intervals at different locations. The bunch weight was recorded on the first, second, third, and fourth days, and it was noticed that a significantly higher PLW was recorded in bunches taken from the untreated control plants as compared to the other treatments. On the third day of storage, PLW in control reached up to 5.80%, whereas PLW in Bio-Pulse^®^/sulfur was significantly lower (3.49%). On the fourth day, PLW of untreated control had the highest value of 7.23%. However, the PLW value in Bio-Pulse^®^/sulfur treatment was only 5.08, which was on par with Eco-Pesticide^®^/sulfur treatment (5.10%) at ICAR-NRCG. A more or less similar trend was recorded at the other three locations, namely, MRDBS and farmers' plots at Narayangaon and Junnar in the Pune district of Maharashtra (Table 4).

**Table 4:** Effect of biocontrol agent formulations on the shelf life of bunches at different locations.

**Treatments**	**Physiological loss in weight (%)**				**No. of rotten berries**	**No. of fallen berries**
	**Day 1**	**Day 2**	**Day 3**	**Day 4**		
**At ICAR-NRCG, Pune**						
T_1_-Eco-Pesticide	1.91 (7.91)	3.57 (10.87)	4.74 (12.57)	5.44 (13.49)	1.50	4.75
T_2_-Bio-Pulse	1.81 (7.70)	3.10 (10.13)	4.70 (12.50)	5.33 (13.34)	1.75	5.25
T_3_-Bio-Care 24	1.86 (7.84)	3.93 (11.42)	4.78 (12.60)	5.45 (13.49)	1.25	4.25
T_4_-Eco-Pesticide/sulfur	1.51 (7.02)	3.36 (10.55)	3.59 (10.91)	5.10 (13.05)	1.25	3.50
T_5_-Bio-Pulse/sulfur	1.61 (7.29)	2.18 (8.48)	3.49 (10.76)	5.08 (13.01)	1.50	3.50
T_6_-Bio-Care 24/sulfur	1.78 (7.65)	3.57 (10.88)	3.87 (11.32)	5.30 (13.30)	0.75	3.00
T_7_-Sulfur 80%WDG	1.61 (7.29)	2.32 (8.73)	3.35 (10.53)	5.06 (12.97)	0.25	5.75
T_8_-Water control	1.84 (7.76)	3.29 (10.44)	5.51 (13.57)	6.88 (15.20)	3.25	6.00
T_9_-Untreated control	2.20 (8.52)	3.29 (10.44)	5.80 (13.92)	7.23 (15.58)	4.00	6.25
**CD (P** **=** **0.05)**	**0.81**	**0.70**	**0.87**	**0.83**	**1.72**	**NA**
**At MRDBS, Pune**						
T_1_-Eco-Pesticide	1.87 (7.85)	3.64 (11.00)	4.81 (12.67)	5.44 (13.49)	3.00	3.25
T_2_-Bio-Pulse	1.52 (7.07)	3.19 (10.28)	4.74 (12.57)	5.34 (13.36)	2.25	3.25
T_3_-Bio-Care 24	2.20 (8.51)	3.99 (11.51)	3.99 (11.79)	5.52 (13.59)	1.50	3.50
T_4_-Eco-Pesticide/sulfur	1.53 (7.10)	3.42 (10.66)	3.57 (10.89)	5.33 (13.35)	1.50	3.25
T_5_-Bio-Pulse/sulfur	1.90 (7.92)	2.33 (8.78)	3.62 (11.41)	5.15 (13.11)	1.50	2.75
T_6_-Bio-Care 24/sulfur	1.91 (7.95)	3.65 (11.01)	3.91 (11.74)	5.18 (13.15)	0.75	2.75
T_7_-Sulfur 80%WDG	1.82 (7.74)	2.38 (8.87)	3.40 (11.26)	5.55 (13.62)	0.25	3.25
T_8_-Water control	1.65 (7.36)	3.39 (10.60)	5.62 (13.70)	6.92 (15.25)	5.50	4.25
T_9_-Untreated control	2.16 (8.44)	3.97 (11.49)	5.88 (14.03)	7.45 (15.83)	5.75	4.50
**CD (P** **=** **0.05)**	**0.47**	**0.34**	**0.74**	**0.20**	**2.12**	**NA**
**At Narayangaon, Pune**						
T_1_-Eco-Pesticide	1.87 (7.85)	3.64 (10.98)	4.81 (12.66)	5.44 (13.48)	2.75	3.25
T_2_-Bio-Pulse	1.50 (7.03)	3.17 (10.24)	4.76 (12.59)	5.39 (13.41)	3.00	2.25
T_3_-Bio-Care 24	2.19 (8.50)	3.87 (11.34)	4.85 (12.72)	5.55 (13.62)	3.50	2.25
T_4_-Eco-Pesticide/sulfur	1.52 (7.08)	3.41 (10.63)	3.59 (10.91)	5.43 (13.47)	2.00	2.25
T_5_-Bio-Pulse/sulfur	1.89 (7.89)	2.34 (8.79)	3.81 (11.25)	5.21 (13.19)	2.00	1.75
T_6_-Bio-Care 24/sulfur	1.92 (7.96)	3.67 (11.04)	3.94 (11.44)	5.24 (13.23)	1.25	1.75
T_7_-Sulfur 80%WDG	1.54 (7.11)	2.35 (8.82)	3.42 (10.66)	5.62 (13.70)	0.75	3.50
T_8_-Water control	1.68 (7.44)	3.38 (10.59)	5.77 (13.90)	6.94 (15.27)	3.75	2.25
T_9_-Untreated control	2.17 (8.45)	4.10 (11.68)	6.01 (14.18)	7.63 (16.03)	4.50	3.50
**CD (P** **=** **0.05)**	**0.40**	**0.37**	**0.38**	**0.53**	**2.00**	**NA**
**At Junnar, Pune**						
T_1_-Eco-Pesticide	1.90 (7.91)	3.60 (10.93)	4.79 (12.64)	5.46 (13.51)	2.25	2.50
T_2_-Bio-Pulse	1.54 (7.10)	3.15 (10.22)	4.73 (12.53)	5.37 (13.39)	2.50	3.00
T_3_-Bio-Care 24	2.24 (8.55)	3.95 (11.45)	4.80 (12.62)	5.49 (13.54)	2.25	3.00
T_4_-Eco-Pesticide/sulfur	1.85 (7.78)	3.40 (10.61)	3.55 (10.85)	5.15 (13.11)	1.75	3.00
T_5_-Bio-Pulse/sulfur	1.93 (7.96)	2.30 (8.71)	3.53 (10.81)	5.30 (13.30)	1.50	3.00
T_6_-Bio-Care 24/sulfur	1.93 (7.99)	3.64 (11.00)	3.89 (11.35)	5.06 (13.00)	0.75	2.50
T_7_-Sulfur 80%WDG	1.80 (7.70)	2.35 (8.81)	3.38 (10.57)	5.40 (13.43)	0.50	4.00
T_8_-Water control	1.63 (7.33)	3.35 (10.54)	5.56 (13.63)	6.85 (15.16)	2.50	4.25
T_9_-Untreated control	2.15 (8.41)	3.95 (11.46)	5.85 (13.99)	7.27 (15.63)	3.50	4.25
**CD (*****P** **=*** **0.05)**	**NA**	**0.63**	**0.84**	**0.51**	**2.05**	**NA**

*Figures in parenthesis indicate arcsine transformed averages*.

In the case of rotten berries, significant differences were observed among all the treatments. All the treatments with microbial inoculants showed a significantly less number of rotten berries as compared to the untreated control (4.00) and those under water treatment (3.25). The check fungicide sulfur showed minimum rotten berries (0.25) followed by Bio-Care^®^/sulfur (0.75), Eco-Pesticide^®^/sulfur (1.25), and Bio-Care^®^ alone (1.25) at ICAR-NRCG (Table 4). These values were slightly higher at MRDBS, Pune, where the average number of rotten berries in the untreated control was 5.75, followed by those under water treatment (5.50), while sulfur showed the minimum rotten berries (0.25). A more or less similar trend with a slight difference in the number of rotten berries was recorded at the other two locations, namely, farmers' plots at Narayangaon and Junnar in the Pune district of Maharashtra (Table 4).

When comparing the average number of fallen berries among the treatments, the differences were nonsignificant. Among all the treatments, treatment with Bio-Care^®^/sulfur (3.00) showed the minimum fallen berries followed by Bio-Pulse^®^/sulfur (3.50) and Eco-Pesticide^®^/sulfur (3.50) as compared to the water-treated (6.00) and untreated control plants (6.25). Moreover, the average number of fallen berries was also lower in the treatments with individually inoculated plants (Eco-Pesticide: 4.75, Bio-Pulse: 5.25, and Bio-Care: 4.25) as compared to the untreated control (6.25) and even sulfur-treated plants (fungicide check) (5.75) at ICAR-NRCG (Table 4). A similar trend with different values was recorded at MRDBS and farmers' plots at Narayangaon and Junnar in the Pune district of Maharashtra (Table 4). Results indicated that microbial inoculation played an important role in controlling berry rotting as well as berry dropping across the locations.

### Effect of Microbial Bioformulations on Qualitative Parameters of Grapes

Grape quality parameters are the primary determinants of the wine quality. Therefore, the quality of grapes is of utmost importance to the wine industry. The berry quality as affected by bioformulation application was assessed, and the data on observations related to berry quality were recorded. This study suggests that all the bioformulations tested enhanced the shelf life and berry quality significantly. The effects of microbial inoculation, singly or in combination with sulfur, on qualitative parameters like pH, TSS, acidity, berry diameter, and berry length were significantly varied, except for the pH of the grapes at different locations. In the case of pH, no significant difference was observed among different treatments and the untreated control (Table 5). The results of this study authenticate a positive role of the microbial inoculation on the accumulation of TSS and treatment with Bio-Pulse^®^/sulfur exhibited significantly highest TSS (21.88 Brix) followed by Eco-Pesticide^®^/sulfur (20.55 Brix) and Bio-Care^®^/sulfur (19.73 Brix) as compared to the individual inoculation of Bio-Pulse^®^ (19.95 Brix) Bio-Care^®^ (18.95 Brix), Eco-Pesticide^®^ (18.80 Brix), and untreated control plants (16.13 Brix) at ICAR-NRCG (Table 5). In general, plants treated with the newly developed bioformulation showed significantly higher TSS than the check fungicide, sulfur (18.55 Brix). A slight difference in the TSS was recorded at the other three centers/locations. However, the trends were more or less similar.

**Table 5:** Effect of bio-formulations on qualitative parameters of grapes at different locations.

**Treatments**	**pH**	**TSS (Brix)**	**Acidity (%)**	**Berry diameter (mm)**	**Berry length (mm)**
**At ICAR-NRCG, Pune**					
T_1_-Eco-Pesticide	3.16	18.80	0.51 (4.09)	14.85	22.28
T_2_-Bio-Pulse	3.34	19.95	0.47 (3.91)	12.72	22.18
T_3_-Bio-Care 24	3.18	18.95	0.55 (4.23)	12.62	22.10
T_4_-Eco-Pesticide/sulfur	3.15	20.55	0.48 (3.97)	15.70	24.58
T_5_-Bio-Pulse/sulfur	3.45	21.88	0.42 (3.70)	17.49	25.47
T_6_-Bio-Care 24/sulfur	3.36	19.73	0.51 (4.08)	15.11	22.87
T_7_-Sulfur 80%WDG	3.41	18.55	0.52 (4.11)	12.50	23.19
T_8_-Water control	3.45	16.65	0.71 (4.83)	10.31	21.63
T_9_-Untreated control	3.61	16.13	0.72 (4.87)	9.25	21.60
**CD (*****P** **=*** **0.05)**	**NA**	**0.84**	**0.18**	**1.29**	**1.67**
**At MRDBS, Pune**					
T_1_-Eco-Pesticide	3.16	19.25	0.83 (1.06)	17.15	22.33
T_2_-Bio-Pulse	3.34	19.28	0.91 (0.83)	17.48	23.25
T_3_-Bio-Care 24	3.19	19.73	0.80 (1.02)	17.45	22.28
T_4_-Eco-Pesticide/sulfur	3.16	20.55	0.75 (1.00)	18.35	23.78
T_5_-Bio-Pulse/sulfur	3.46	21.88	0.71 (0.80)	18.75	24.28
T_6_-Bio-Care 24/sulfur	3.37	19.95	0.76 (0.76)	18.33	23.20
T_7_-Sulfur 80%WDG	3.43	18.55	0.99 (0.71)	16.95	21.63
T_8_-Water control	3.45	18.40	1.02 (0.91)	16.75	21.58
T_9_-Untreated control	3.59	18.38	1.06 (0.75)	15.60	21.03
**CD (*****P** **=*** **0.05)**	**NA**	**0.83**	**0.08**	**1.38**	**1.60**
**At Narayangaon, Pune**					
T_1_-Eco-Pesticide	3.54	18.05	0.83 (5.22)	17.45	22.28
T_2_-Bio-Pulse	3.50	18.93	0.80 (5.13)	17.48	23.25
T_3_-Bio-Care 24	3.47	18.00	0.83 (5.21)	17.15	22.33
T_4_-Eco-Pesticide/sulfur	3.61	19.92	0.75 (4.96)	18.33	23.78
T_5_-Bio-Pulse/sulfur	3.50	20.67	0.71 (4.84)	18.40	24.28
T_6_-Bio-Care 24/sulfur	3.52	19.12	0.76 (5.00)	18.35	23.28
T_7_-Sulfur 80%WDG	3.50	17.36	0.99 (5.72)	16.95	21.63
T_8_-Water control	3.65	16.85	1.02 (5.80)	16.23	21.58
T_9_-Untreated control	3.62	16.18	1.06 (5.91)	16.00	19.88
**CD (*****P** **=*** **0.05)**	**NA**	**1.60**	**0.23**	**0.89**	**1.29**
**At Junnar, Pune**					
T_1_-Eco-Pesticide	3.34	17.18	0.91 (5.47)	17.48	22.28
T_2_-Bio-Pulse	3.41	18.10	0.80 (5.13)	17.15	23.25
T_3_-Bio-Care 24	3.36	17.33	0.83 (5.22)	17.45	22.33
T_4_-Eco-Pesticide/sulfur	3.45	18.75	0.75 (4.96)	18.35	23.78
T_5_-Bio-Pulse/sulfur	3.61	20.25	0.71 (4.84)	18.40	24.28
T_6_-Bio-Care 24/sulfur	3.45	18.33	0.76 (5.00)	18.33	23.28
T_7_-Sulfur 80%WDG	3.18	16.93	0.99 (5.72)	16.95	21.63
T_8_-Water control	3.16	16.78	1.02 (5.80)	16.75	21.58
T_9_-Untreated control	3.15	16.00	1.06 (5.91)	15.85	20.88
**CD (*****P** **=*** **0.05)**	**NA**	**0.81**	**0.23**	**1.23**	**1.27**

*Figures in parenthesis indicate arcsine transformed averages*.

The percent acidity differed significantly among the treatments. The treatment with Bio-Pulse^®^/sulfur showed significantly lower acidity (3.70%) than the untreated control (4.87%) and water-treated ones (4.83), which were followed by Eco-Pesticide^®^/sulfur (3.97) and Bio-Care^®^/sulfur (4.08) at ICAR-NRCG. A similar trend was recorded at the other three locations (Table 5). Similar to the TSS and percent acidity, the berry diameter and berry length also significantly varied in microbial-inoculated plants and the untreated control plants. Interestingly, maximum berry diameter and berry length were recorded in the plants treated with Bio-Pulse^®^/sulfur across the locations, which was significantly higher than the sulfur alone-treated and untreated control plants (Table 5). The results obtained from ICAR-NRCG, Bio-Pulse^®^/sulfur showed the highest berry diameter (17.49 mm) as compared to the untreated control (9.25 mm). Eco-Pesticide^®^/sulfur and Bio-Care^®^/sulfur were on par with each other and were the second best among all the treatments with berry diameters of 15.70 mm and 15.11 mm, respectively. In the case of berry length, Bio-Pulse^®^/sulfur showed the highest berry length (25.47 mm) as compared to the untreated control (21.60 mm), which was on par with treatment Eco-Pesticide^®^/sulfur having a berry length of 24.58 mm (Table 5). Furthermore, individual inoculation of either of the microbial formulation showed significantly increased berry diameter and berry length across the locations.

### Effect of Microbial Bioformulations on Marketable Yield of Grapes

Fruit yield per plant was recorded, and it was converted to the fruit yield per unit area (q/ha). In general, results showed that all the treatments with newly developed bioformulation increased the yield (kg/vine) significantly as compared to the untreated control. Furthermore, the yield was significantly increased after the application of sulfur in combination with microbial inoculant as compared to the solo bioformulations. Among all the treatments except check fungicide sulfur, Bio-Pulse^®^/sulfur treatment showed the highest yield per vine, which was on par with the treatment Eco-Pesticide^®^/sulfur. On the contrary, the untreated control gave the lowest values on this parameter, while treatments Bio-Care^®^/sulfur recorded the second highest values of yield per vine (Table 6). When compared with the yield obtained from the untreated control, 2.5–3 times more yield was recorded in the plants treated with either of the biopesticides along with sulfur. Even in the case of individual inoculation, the yield per vine was approximately two times higher than the untreated control and water-treated plants across the locations (Table 6).

**Table 6:** Effect of biocontrol agent formulations on marketable yield of grapes at different locations.

**Treatments**	**NRCG, Pune**		**MRDBS, Pune**		**Narayangaon**		**Junnar**	
	**Kg/vine**	**Q/ha**.	**Kg/vine**	**Q/ha**.	**Kg/vine**	**Q/ha**.	**Kg/vine**	**Q/ha**.
T_1_-Eco-Pesticide	10.27	185.59	10.98	198.46	10.98	198.46	10.98	198.46
T_2_-Bio-Pulse	10.08	182.20	9.55	172.57	10.02	181.16	10.46	189.07
T_3_-Bio-Care 24	8.59	155.28	8.83	159.62	8.59	155.35	8.83	159.62
T_4_-Eco-Pesticide/sulfur	14.94	270.09	14.20	256.65	13.79	249.23	14.15	255.74
T_5_-Bio-Pulse/sulfur	15.02	271.54	15.47	279.70	15.14	273.73	15.97	288.74
T_6_-Bio-Care 24/sulfur	12.73	230.18	12.91	233.32	12.91	233.32	12.91	233.32
T_7_-Sulfur 80%WDG	16.63	300.67	17.93	324.17	18.18	328.69	17.68	319.65
T_8_-Water control	6.44	116.50	5.94	107.44	6.83	123.51	7.18	129.79
T_9_-Untreated control	5.35	96.75	4.26	76.93	5.53	99.89	5.64	101.97
**CD (*****P** **=*** **0.05)**	**1.19**		**2.18**		**2.31**		**2.17**	

## Discussion

The aim of this study was to evaluate the microbe-based technologies, such as Bio-Pulse^®^, Eco-Pesticide^®^, and Bio-Care^®^, developed at ICAR-NBAIM against *Erysiphe necator* causing powdery mildew disease in grapes (*Vitis vinifera* L.). The basic concept behind evaluating these biopesticides in grapes is to reduce the application of chemical fungicides and improve the qualitative parameters in grapes without compromising the yield. As mostly grapes are used for table purposes, which demands them to be free from pesticide residue, the use of chemicals to control the grape diseases becomes an unwarranted practice (Cordero-Bueso et al., 2014; Warneke et al., 2022). To keep this with the consumer expectations, most of the vine industries follow a “zero pesticides” policy promoting viticulture in a more or less fully organic manner (Alori and Babalola, 2018). To determine whether these bio-formulations can be used as an effective technology to control *E. necator* causing powdery mildew in grapes, the effects of microbial inoculants/technologies on *E. necator* were first examined. Furthermore, we examined whether there was a difference in disease severity (PDI) in microbial inoculants-treated vs. sulfur-treated/water-treated/untreated control plants. A comparative analysis was carried out, and the effects of treatments on PDI of powdery mildew on leaves and bunches, physiological weight loss, the average number of rotten berries, the average number of fallen berries, yield, and qualitative parameters in treated berry were recorded. Comparative analyses indicated that on an average, the microbial inoculants significantly controlled spread of the disease, physiological weight loss, the average number of fallen berries, and increased qualitative parameters such as pH, TSS, berry diameter, berry length, and fruit yield in the plants as compared to water-treated and untreated control across the experimental sites. In general, bioinoculants/microbial bioformulations performed better when used in alternation with sulfur as compared to the individual applications. Apart from the check fungicide sulfur, Bio-Pulse/sulfur treatment showed the highest values in terms of disease control which was on par with the treatment Eco-Pesticide/sulfur. In contrast, untreated control showed the highest PDI, while treatment with Bio-Care/sulfur was found second-best treatment across the locations.

Results indicated that these bioformulations/products were found to limit the PDI on leaves and bunches of grapevines effectively with a simultaneous increase in the yield and enhanced quality parameters in grapes. The reduction of PDI of powdery mildew on leaves and bunches was supposed to be either due to the reduction of primary inoculum or controlling the further infection/invasion of the pathogen (Lombardi et al., 2020). The application of bioagents could possibly employ the mechanisms like mycoparasitism, nutrient competition, hyperparasitism, antibiosis, competition for space, and production of cell-wall degrading enzymes (Harman et al., 2004; Robinson-Boyer et al., 2009; Malviya et al., 2020), which could have reduced the invasion of *E. necator*. Since the bioagents performed well upon foliar spray on the leaf surface, it represents a high degree of rhizosphere/phyllosphere competence, which is the first and foremost requirement for developing a successful biocontrol system (Sawant et al., 2012; Pylak et al., 2019; Santos et al., 2021). The bioagents used in this study were earlier reported to induce systemic resistance (ISR) in different crops against plant pathogens (Singh et al., 2016a,b, 2019a,b). In this study, ISR could also be a mechanism for biocontrol of *E. necator*. This induction of ISR by phyllosphere application of biocontrol agents are in line with the findings of Sawant et al. (2020), which indicated that the field application of *Trichoderma* strains induced systemic resistance in grapevines against powdery mildew pathogens. It also acts as an inducer for resistance in treated plants against the target pathogens (Harman et al., 2004; Shoresh et al., 2010; Malviya et al., 2020). It is also clear that they can grow within a wide range of temperature and other environmental conditions (data not shown). The present investigation clearly indicated that the application of microbial bioformulations not only reduces the disease severity on leaves and bunches, but it also reduces the physiological weight loss, berry rotting, and berry dropping in grapes. These are commercially very crucial traits and could significantly affect the yield quality as well as quantity. Furthermore, microbial inoculation also improves the qualitative traits such as TSS, berry diameter, and berry length across the locations as compared to fungicide-treated and untreated control plants. *Trichoderma* has a positive effect on titratable acidity, pH, and TSS of tomato crop; foliar application of *Trichoderma* decreased the acidity and increased the TSS content (Palacios-Torres et al., 2019). It not only increases nutrient absorption capacity (López-Bucio et al., 2015), but may also increase the accumulation of sugars in the fruits (Molla et al., 2012). This is because the application of *Trichoderma* improved the carbohydrate metabolism and increased the accumulation of starches in the plant (Shoresh and Harman, 2008). Lombardi et al. (2020) stated that microbial inoculants highly affected the representation of proteins associated with responses to stress/external stimuli, nutrient uptake, protein metabolism, carbon/energy metabolism, and secondary metabolism, also providing a possible explanation for the presence of specific metabolites in fruits. Several research reports strongly supported that microbial inoculation improves the nutritional quality not only in grapes but also in other crops (Singh et al., 2010, 2016a,b, 2018; Yadav et al., 2022).

Sulfur is an important element with fungicidal properties and is widely used in the management of plant diseases in grapes and powdery mildew in particular. Moreover, sulfur (600 g/100 L) is one of the key fungicides used for the effective management of powdery mildew and is known to improve the grape yield under commercial cultivation (Savocchia et al., 2011; Ahmed, 2018; Essling et al., 2021). In this study, ¾ amount of sulfur is being reduced without compromising the product quality and quantity, which is a significant reduction. Apart from saving the dose of sulfur, it has been shown to have protective rather than curative action as much as chemical management of powdery mildews is concerned. It kills the spores of *Erysiphe necator* and thus protects the vines from new infections (Rantsiou et al., 2020; Sellitto et al., 2021). It does not kill the fungus itself, and the best use of sulfur, therefore, is to prevent vines from becoming infected rather than to suppress the infections once they have developed. Existing mature fungal colonies begin producing more spores a week after a sulfur spray is applied (Konstantinidou-Doltsinis et al., 2007; Cordero-Bueso et al., 2014; Warneke et al., 2022). Thus, combining sulfur application with the biocontrol agents that could reduce the chances of post-application inoculumn buildup would be a better strategy. The study conducted is in line with that, and it is clearly evidenced from the results obtained. In this study, PDI on leaves and bunches was significantly reduced when bioagents were applied with sulfur (Tables 2, 3). Reduction in rotten and fallen berries by biocontrol agents + sulfur application (Table 4) indicated that the BCA could reduce the persistent fungal mycelia from the infected vines, which sulfur alone could not be performed at the same doses. This also has a direct impact on the shelf life of the berries, which could have been clearly made out from the results (Table 4). The results are in line with the findings of Sawant and Sawant (2010) and Ahmed (2018). Apart from supplementing the sporicidal properties of sulfur, the application of bioagents also has effects on plant growth induction. Suppression of disease and consequent improvement in growth could be one of the reasons for improved berry quality and yield.

The improvement in the yield and yield performance are in line with the field study conducted by Tesfagiorgis et al. (2014), where through the application of biocontrol agents and silicon, 10–70% of disease reduction was obtained. Reduction in the disease and improvement in yield parameters as obtained in the present investigation is also significant from the fact that fungicidal resistance is building-up in powdery mildew fungi (Vielba-Fernández et al., 2020), thus bioagents with good field bio-efficacy should be widely tested and adopted for sustainable grape farming. Shelf life of bunches was significantly improved upon inoculation of biocontrol agents, and the effects were more prominent with biocontrol agents + sulfur application. These results are in line with the reports by Sawant et al. (2017), where improvement in berry shelf life from *Trichoderma* application was reported from the field trials. Since improved shelf life has a direct correlation with the market value of berries, the application of Eco-Pesticide + sulfur and Bio-Pulse + sulfur could increase the benefit–cost ratio for grape cultivation. Furthermore, microbial inoculation significantly increased the grape yield (kg/vine) by 2- to 3-fold as compared to the untreated control under pathogenic stress of *E. necator* across the locations. These results are in agreement with the other researchers who reported that microbial inoculants have a positive impact on the yield of grapes grown under the pathogenic stress of *E. necator* (El-Mogy, 2017; Johnston-Monje et al., 2021; Laurent et al., 2021). Dario et al. (2008) stated that commercial formulations of *Bacillus subtilis*, namely, Serenade and Milastin K, showed effective and consistent suppression of *E. necator* under greenhouse and field conditions. Milastin K when used in alternation with fungicides performed best in disease control and increasing yield of grapevine (Dario et al., 2008; Sawant et al., 2011). Furthermore, *Ampelomyces quisqualis*, a typical biocontrol agent for control of powdery mildew also functions better with sulfur (unpublished data but paper accepted). Hence, combined application gives better results, and it is also preferred in integrated disease management under an organic production system (Sawant et al., 2011, 2017). In the eventuality of not obtaining the required level of disease control by the application of microbial formulations alone, a need-based application of fungicide is needed (Tesfagiorgis et al., 2014).

## Conclusion

It has been observed that application of microbe-based technologies/bioformulations individually or in combination with sulfur significantly decreased powdery mildew disease on leaves and bunches and increased the quality parameters in grapes under this pathogenic stress. Microbe-based technologies, such as Eco-pesticide^®^, Bio-Pulse^®^, and Bio-Care 24^®^, emerge as promising biopesticides for managing powdery mildew at every stage of grapevine, which can be further maintained by combining sulfur in a cooperative manner under severe infections. It was also found that application of either of the biopesticides alone or in combination with sulfur significantly suppresses disease development and reduces PDI in a cooperative manner and saves the plants from fungal infection. It was also noticed that plants treated with Eco-pesticide^®^, Bio-Pulse^®^, and Bio-Care 24^®^ exhibit significant enhancement in the nutritional quality of grapes. These microbial technologies also increased marketable yield per plant enhancing the crop economy in the favor of the grower/farmer. With the help of the findings of this investigation, we conclude that microbe-based technologies could be a potential alternative of toxic chemical fungicides and can be applied at a larger scale to control powdery mildew disease in grapes.

## Data Availability Statement

The original contributions presented in the study are included in the article/supplementary material, further inquiries can be directed to the corresponding author/s.

## Author Contributions

US, AS, and SS: conceptualization. DM and RT: methodology. DM, RT, NK, SP, and SS: validation. US and SS: formal analysis and supervision. DM, RT, and SS: investigation. AS and RS: resources. SS: data curation. US, HS, and JR: writing—original draft preparation and writing—review and editing. US and AS: project administration. US: funding acquisition. All authors have read and agreed to the published version of the manuscript.

## Funding

This research was funded by the Network Project on Application of Microorganisms in Agriculture and Allied Sectors (AMAAS), Indian Council of Agricultural Research, Department of Agriculture, Research and Education, Ministry of Agriculture and Farmer Welfare, Government of India, New Delhi.

## Conflict of Interest

The authors declare that the research was conducted in the absence of any commercial or financial relationships that could be construed as a potential conflict of interest.

## Publisher's Note

All claims expressed in this article are solely those of the authors and do not necessarily represent those of their affiliated organizations, or those of the publisher, the editors and the reviewers. Any product that may be evaluated in this article, or claim that may be made by its manufacturer, is not guaranteed or endorsed by the publisher.
